# Enhanced Diagnostic Accuracy for Septic Arthritis Through Multivariate Analysis of Serum and Synovial Biomarkers

**DOI:** 10.3390/jcm14155415

**Published:** 2025-08-01

**Authors:** Hyung Jun Park, Ji Hoon Jeon, Juhyun Song, Hyeri Seok, Hee Kyoung Choi, Won Suk Choi, Sungjae Choi, Myung-Hyun Nam, Dong Hun Suh, Jae Gyoon Kim, Dae Won Park

**Affiliations:** 1 Department of Orthopedic Surgery, Korea University Ansan Hospital, Korea University College of Medicine, Ansan 15355, Republic of Korea; ppakyung@hotmail.com (H.J.P.); dsuh@korea.ac.kr (D.H.S.); 2 Department of Thoracic and Cardiovascular Surgery, Samsung Medical Center, Sungkyunkwan University School of Medicine, Seoul 06351, Republic of Korea; jjhinlove@naver.com; 3 Department of Emergency Medicine, Korea University Anam Hospital, Korea University College of Medicine, Seoul 02841, Republic of Korea; songcap97@hotmail.com; 4 Division of Infectious Diseases, Korea University Ansan Hospital, Korea University College of Medicine, Ansan 15355, Republic of Korea; hyeri.seok@gmail.com (H.S.); cmcws@hanmail.net (W.S.C.); 5 Division of Infectious Diseases, National Health Insurance Service Ilsan Hospital, Goyang 10444, Republic of Korea; hkchoi0000@naver.com; 6 Family Medicine, Providence St. Joseph Hospital, Eureka, CA 95501, USA; csjmd888@gmail.com; 7 Department of Laboratory Medicine, Korea University Anam Hospital, Korea University College of Medicine, Seoul 02841, Republic of Korea; yuret@korea.ac.kr

**Keywords:** septic arthritis, biomarkers, C-reactive protein, pentraxin 3, presepsin, interleukin 6, white blood cells, diagnosis

## Abstract

**Background:** Septic arthritis is an orthopedic emergency. However, optimal biomarkers and diagnostic criteria remain unclear. The study aimed to evaluate the diagnostic performance of routinely used and novel biomarkers, including serum C-reactive protein (CRP), synovial white blood cells (WBC), pentraxin-3 (PTX3), interleukin-6 (IL-6), and presepsin, in distinguishing septic from non-septic arthritis. **Methods:** Thirty-one patients undergoing arthrocentesis were included. Patients were categorized into septic and non-septic arthritis groups. Synovial fluid and serum samples were analyzed for five biomarkers. Diagnostic performance was assessed by calculating the area under the curve (AUC), accuracy, sensitivity, specificity, positive predictive value (PPV), and negative predictive value (NPV). **Results:** Synovial WBC demonstrated the highest diagnostic performance among single biomarkers (AUC = 0.837, *p* = 0.012). Among novel biomarkers, PTX3 showed the highest accuracy and sensitivity. The serum CRP and synovial WBC combination yielded an AUC of 0.853, with 100% sensitivity, 68.0% specificity, 42.9% PPV, and 100% NPV. Adding all three novel biomarkers to this combination increased the AUC to 0.887 (*p* = 0.004), maintaining 100% sensitivity and NPV. When individually added, PTX3 achieved 100% sensitivity and NPV, while presepsin showed the highest specificity (96.0%), PPV (75.0%), and accuracy (87.1%). **Conclusions:** Serum CRP and synovial WBC remain essential biomarkers for diagnosing septic arthritis; however, combining them with PTX3, IL-6, and presepsin improved diagnostic accuracy. PTX3 is best suited for ruling out septic arthritis due to its high sensitivity and NPV, whereas presepsin is more useful for confirmation, given its specificity and PPV. These results support a tailored biomarker approach aligned with diagnostic intent.

## 1. Introduction

Diagnosing septic arthritis is a significant challenge for clinicians. Recognized as an orthopedic emergency, septic arthritis needs urgent diagnosis and treatment owing to its potential for severe morbidity and mortality [1,2,3,4]. If treatment is delayed, it can lead to irreversible damage to the intra-articular structure, resulting in functional disability and life-threatening conditions in 5–15% of patients [5,6,7,8]. Therefore, early diagnosis is crucial for the prognosis of patients with septic arthritis. However, distinguishing the clinical features of septic arthritis from those of non-septic arthritis, such as degenerative, gouty, and other inflammatory arthritis, remains difficult [1]. The gold standard for diagnosing septic arthritis is synovial culture; however, its sensitivity is not high, and the results take several days [9]. Alternatively, synovial fluid analysis, including white blood cells (WBCs) and polymorphonuclear leukocytes (PMNs) percent, has been performed as an initial diagnostic criterion [1,10]. Serum WBC and C-reactive protein (CRP) levels are also used to evaluate the severity of systemic infection [1,2,11]. However, among these performed laboratory tests, there was no consensus on which biomarkers yielded the most significant diagnostic value in patients with suspected septic arthritis [12,13,14].

Recently, novel biomarkers have been proposed as diagnostic tools for septic arthritis [15,16,17,18,19,20,21]. Pentraxin 3 (PTX3), interleukin 6 (IL-6), and presepsin are among these biomarkers [17,18,22]. PTX3, one of the acute phase proteins like CRP, has excellent diagnostic performance in detecting periprosthetic joint infection (PJI) [18]. IL-6, a pro-inflammatory cytokine, plays a central role in the early phase of inflammation [17]. In contrast, presepsin is released during the phagocytosis of bacteria by monocytes and reflects a different stage in the host immune response [22]. Given these differences, each biomarker reflects a distinct aspect or temporal phase of the infection cascade. Although synovial biomarkers generally show higher accuracy than serum markers in diagnosing septic arthritis, no consensus has been established regarding the optimal synovial biomarkers for diagnosing septic arthritis [15,17,18,19,20]. Furthermore, most previous studies have focused on the diagnostic performance of single biomarkers through univariate analysis [15,16,17,18,19,20]. However, considering the diverse pathophysiological origins, kinetics, and immunological roles of these markers, combining multiple biomarkers may offer a more comprehensive diagnostic approach. Despite this theoretical advantage, few studies have evaluated the diagnostic potential of such combinations in clinical settings.

We aimed to determine the diagnostic performance of serum and synovial fluid biomarkers in detecting septic arthritis and identify the optimal combination of these biomarkers. We hypothesized that the novel synovial biomarkers have greater diagnostic value than the routinely used biomarkers. Additionally, we hypothesized that combining these biomarkers would yield superior diagnostic performance compared with a single biomarker alone.

## 2. Material and Methods

### 2.1. Study Design and Population

**Figure 1:**
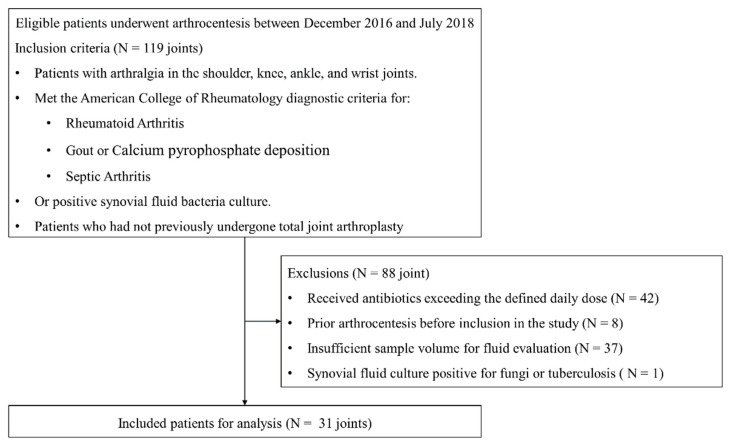
Flowchart of the study population.

This study was a prospective investigation that included patients who underwent arthrocentesis between December 2016 and July 2018, all of whom provided informed consent. Inclusion criteria were as follows: patients with shoulder, knee, ankle, or wrist arthralgia who met the American College of Rheumatology diagnostic criteria for rheumatoid arthritis, gout, or septic arthritis, and had no history of prior total joint arthroplasty. Among these eligible patients, 88 were excluded based on predefined criteria: 42 had received antibiotics exceeding the defined daily dose, 8 had undergone arthrocentesis before study inclusion, and 37 had an insufficient sample volume for fluid analysis. Additionally, prosthetic joints and immunocompromised status (e.g., human immunodeficiency virus infection) were designated as exclusion criteria; however, no such cases were identified among the enrolled patients. One patient whose synovial fluid culture was positive for non-bacterial organisms, such as fungi or tuberculosis, was excluded from the study. As a result, thirty-one patients were ultimately included in the study (Figure 1).

The included patients were divided into two groups (septic arthritis and non-septic arthritis) based on clinical, radiographic, microbiological culture, and laboratory results [6]. In this prospective observational study, the diagnostic criteria for septic arthritis were established before patient enrollment. Patients were to be classified as having septic arthritis if synovial fluid culture yielded a bacterial isolate [21]. Alternatively, in culture-negative cases, a diagnosis could be rendered when at least three of four independent specialists (two infectious disease physicians, one orthopedic surgeon, and one rheumatologist) reached a consensus based on clinical presentation, laboratory parameters, and radiographic evidence [21]. Among 31 patients, six were allocated to the septic arthritis group and 25 to the non-septic arthritis group. In the septic arthritis group, the most common pathogen identified was *Staphylococcus aureus* (N = 3), followed by *Staphylococcus epidermidis* (N = 1), *Streptococcus pyogenes* (N = 1), and *Klebsiella pneumoniae* (N = 1). In the non-septic arthritis group, the underlying conditions included gout or calcium pyrophosphate deposition (N = 10), rheumatoid arthritis (N = 1), synovitis associated with ankylosing spondylitis (N = 1), and acute exacerbation of osteoarthritis (N = 9), as well as post-traumatic arthritis (N = 1). Additionally, three cases in which septic arthritis could not be initially excluded were ultimately classified as non-septic following multidisciplinary clinical discussion. The mean age of these patients was 58.5 ± 17.7 years, and the mean body mass index (BMI) was 24.7 ± 3.3 kg/m^2^. Although a height difference was observed between the groups, no differences were found in other baseline characteristics, including BMI (Table 1). The study was approved by our institutional review board (2016AS0037).

### 2.2. Laboratory Evaluation

All patients underwent arthrocentesis for synovial fluid collection, and whole blood was sampled to evaluate serum biomarkers such as WBC, CRP, and erythrocyte sedimentation rate during the initial evaluation. Synovial fluid analysis was performed to determine the WBC count and the percentage of PMNs. Synovial fluid culture and fluid examination under polarized light microscopy were conducted according to our hospital’s protocol. The remaining synovial fluid was centrifuged at 250 rpm for 20 min at 4 °C. The supernatants were collected, frozen, and stored at −80 °C until testing [23]. Synovial fluid PTX3 and IL-6 levels were measured using commercially available enzyme-linked immunosorbent assays (R&D Systems, Minneapolis, MN, USA). Presepsin levels in synovial fluid were measured using a Pathfast^®^ immunoanalyzer presepsin kit (Mitsubishi Chemical Corporation, Tokyo, Japan). Values below the detection limits of each biomarker were recorded as zero.

### 2.3. Statistical Analysis

To ensure the reliability of group classification, inter-rater agreement among the four independent reviewers was assessed using kappa statistics. Kappa value was 0.837, indicating a very high level of agreement among the four raters [24]. Mann–Whitney and Fisher’s exact tests and linear-by-linear association analysis were used to compare the characteristics of the patients included. The area under the curve (AUC) was measured to compare the diagnostic performance of each biomarker in combination. Receiver operating characteristic curve analysis with the Youden index was utilized to determine the cutoff value for differentiating septic from non-septic arthritis. All data were analyzed using SPSS version 20 (IBM Corp., Armonk, NY, USA), with *p* < 0.05 considered statistically significant. The statistical power to differentiate septic and non-septic arthritis using one of the novel biomarkers (PTX3) was 0.95. Power analysis was performed using G*Power version 3.1.9.7 (Heinrich-Heine-University, Düsseldorf, Germany).

## 3. Results

**Figure 2:**
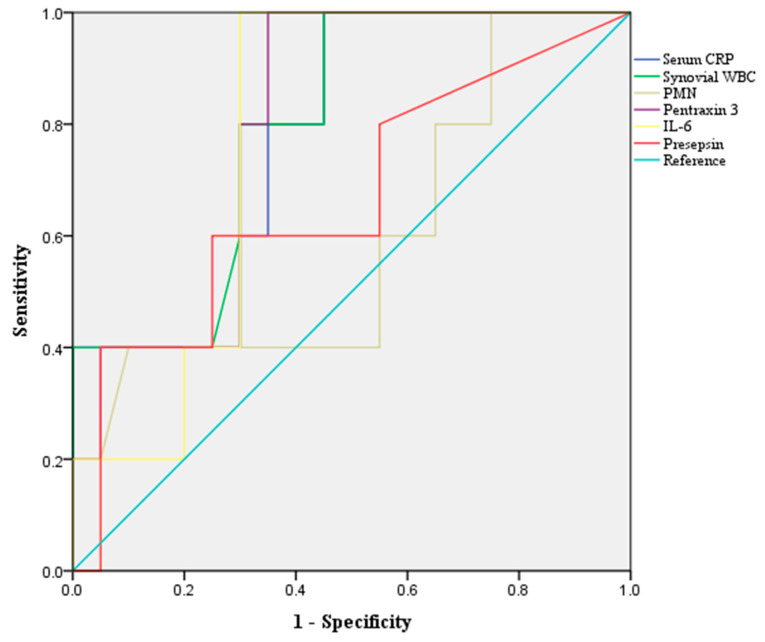
Receiver operating characteristic curves for single biomarkers in diagnosing septic arthritis.

Synovial WBC had the most significant value for detecting septic arthritis in a single test. The serum CRP, synovial WBC, and PTX3 levels were significantly higher in the septic arthritis group than in the non-septic arthritis group (*p* = 0.022, *p* = 0.023, and *p* = 0.017, respectively, Table 2). The mean synovial WBC count was 72,028.6 ± 59,222.3 cells/μL in the septic group and 25,042.8 ± 26,784.2 cells/μL in the non-septic group (*p* = 0.023, Table 2). Although the mean PTX3 was 366.7 ± 618.0 ng/mL in the septic group and 53.6 ± 99.3 ng/mL in the non-septic group (*p* = 0.017), other novel biomarkers, such as IL-6 and presepsin, did not show significant differences between the two groups (*p* = 0.227 and *p* = 0.053, respectively, Table 2). The diagnostic performance of each biomarker was evaluated by calculating the AUC (Figure 2). The AUC for synovial WBC was 0.837 (95% confidence interval [CI], 0.678–0.995; *p* = 0.012, Table 3, Figure 2). When a cutoff value was set at 34,200.0 cells/μL, the accuracy was 77.4%, with a sensitivity of 83.3% and a specificity of 76.0%. Although the AUC for PTX3 was lower than that for serum CRP (0.813 and 0.817, respectively), PTX3 showed a higher accuracy than serum CRP (74.2% and 71.0%, respectively, Table 3). However, IL-6 and presepsin had lower AUCs than serum CRP and synovial WBC (Table 3).

**Figure 3:**
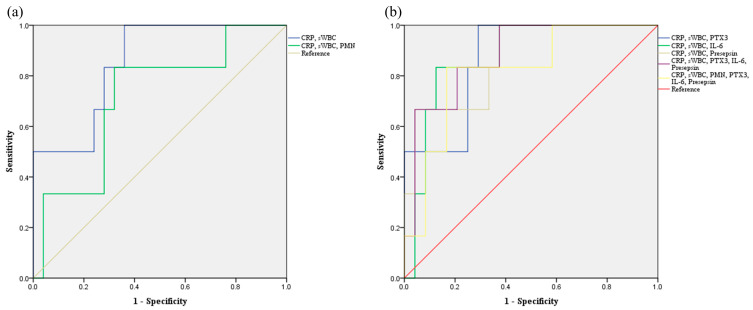
Receiver operating characteristic curves for combined biomarkers in diagnosing septic arthritis. (**a**) Combination of routinely used biomarkers. (**b**) Combination of routinely used and novel biomarkers.

**Figure 4:**
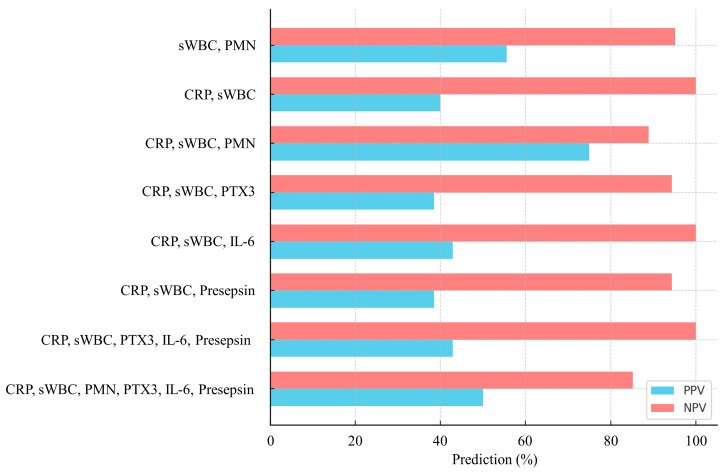
Positive and negative predictive values by biomarker combination.

Combining serum CRP and synovial WBC with novel biomarkers enhanced diagnostic performance, with the optimal combination varying according to the specific biomarker used (Figure 3). The combination of serum CRP and synovial WBC yielded an AUC of 0.853 (95% CI, 0.702–1.000; *p* = 0.008), with an accuracy of 74.2%, sensitivity of 100%, specificity of 68.0%, PPV of 42.9%, and NPV of 100%, respectively (Table 4, Figure 3 and Figure 4). When all three novel biomarkers (PTX3, IL-6, and presepsin) were added, the AUC increased to 0.887 (95% CI, 0.755–1.000; *p* = 0.004), while maintaining 100% sensitivity and NPV, with a specificity of 64.0%, PPV of 40%, and accuracy of 71.0% (Table 4). When individual novel biomarkers were added to the CRP and synovial WBC combination, diagnostic performance varied. Presepsin achieved the highest specificity (96.0%) and accuracy (87.1%), with a PPV of 75.0%, although sensitivity decreased to 50.0% and NPV to 88.9%. PTX3 maintained 100% sensitivity and NPV, with a specificity of 68.0%, PPV of 42.9%, and accuracy of 74.2%. IL-6 demonstrated similar findings, with a sensitivity of 83.3%, specificity of 68.0%, PPV of 38.5%, NPV of 94.4%, and accuracy of 71.0% (Table 4, Figure 3 and Figure 4).

## 4. Discussion

Septic arthritis is a time-sensitive and potentially life-threatening condition that requires rapid and accurate diagnosis to avoid irreversible joint damage and systemic complications [1,2,3]. Serum and synovial biomarkers, such as serum CRP and synovial WBC, have been used as initial diagnostic criteria; however, the diagnostic accuracy of these biomarkers has limitations [1,2,10,11]. In response, novel biomarkers have been introduced to enhance the accuracy of detecting septic arthritis [15,16,17,18,19,20]. However, no consensus has been reached on which biomarkers provide the highest diagnostic value in diagnosing septic arthritis, and most previous studies focused on single-marker performance and did not sufficiently evaluate the diagnostic value of biomarker combinations [15,17,18,19,20]. The principal finding of our study is that combining routinely used markers with novel biomarkers significantly enhances diagnostic performance, offering tailored diagnostic strategies depending on clinical objectives such as early screening or confirmatory diagnosis.

Our results did not support our hypothesis that the novel synovial biomarkers had greater diagnostic value than the routinely performed laboratory tests. Several biomarkers, including serum CRP, synovial WBCs, and PMNs, have been used for diagnosing septic arthritis [1,10,25]. Numerous studies have investigated the most suitable biomarkers for diagnosis and have sought to determine their optimal cut-off values [1,6,11,12,13,16,26,27]. One study reviewed 719 patients to evaluate the diagnostic potential of serum and synovial fluid biomarkers in diagnosing septic arthritis and PJI [25]. They included CRP and WBC in peripheral blood and WBC, percentage of PMNs, and lactate dehydrogenase in synovial fluid. They reported that serum CRP, synovial WBC, and percentage of PMNs were the most significant diagnostic biomarkers. However, other studies reported that the diagnostic performance of these routinely used biomarkers was not high [13,16]. One study evaluated the effectiveness of synovial biomarkers in 796 children with arthralgia [13]. They found that none of the biomarkers, including synovial WBC, absolute PMN count, percentage of PMNs, glucose, and protein, achieved an AUC > 0.8, indicating insufficient accuracy. This limitation has led to the proposal of novel biomarkers for the accurate diagnosis of septic arthritis. Several biomarkers have been reported, including PTX3, IL-6, and presepsin. One study examined the diagnostic value of PTX3 in both serum and synovial fluid among 128 patients suspected of having PJI [18]. They found that although serum PTX3 failed to differentiate between infected and non-infected conditions, synovial PTX3 demonstrated significant diagnostic potential. Another biomarker, IL-6, had been reported in systematic reviews for its role in diagnosing joint infection, with an AUC value > 0.9 [20,28]. Presepsin was also considered a valuable biomarker for distinguishing septic arthritis from non-septic arthritis [22,29]. One study involving 75 patients evaluated serum and synovial presepsin levels for the diagnosis of septic arthritis. They reported that synovial presepsin had a significant diagnostic value, with an AUC of 0.93 [29]. A systematic review evaluated the diagnostic performance of several novel biomarkers in serum and synovial fluid, including PTX3, but excluding IL-6 and presepsin [13]. They concluded that the most useful biomarker was soluble tumor necrosis factor receptor 2 (sTNF-R2), with an AUC of 1.00 (*p* < 0.001). However, PTX3 also showed a high AUC of 0.94 (95% CI, 0.84–1.00; *p* < 0.001), consistent with our findings. However, in our study, synovial WBCs demonstrated superior diagnostic performance compared with novel biomarkers. This result may be attributed to the higher synovial WBC levels found in the septic arthritis group in our study compared with previous studies [13,17]. If the mean value of a specific variable showed a substantial difference between the two groups, it could be inferred that the variable had a significant diagnostic value for distinguishing between the groups [30]. Dechnik et al. reported that synovial WBCs had limited diagnostic value, with an AUC of ≤0.8, as the mean synovial WBC count in septic arthritis did not exceed 20,000 cells/μL [13]. In contrast, Lenski et al. reported a significant diagnostic value for synovial WBC, with an AUC of 0.850, where the mean synovial WBC in septic arthritis was 43,800 cells/μL [17]. Our study results were aligned with those of Lenski et al., with mean synovial WBCs exceeding 70,000 cells/μL. Meanwhile, IL-6 and presepsin showed lower AUC values than the routinely used biomarkers. Presepsin did not reach statistical significance (*p* = 0.051); however, it showed the highest accuracy among all biomarkers in our study. With a larger sample size, it could have demonstrated significant diagnostic potential as well. Although identifying novel biomarkers, such as synovial PTX3, is essential for diagnosing septic arthritis, the routinely used biomarkers still play a crucial role in detecting septic arthritis. In addition to the biomarkers assessed in this study, several other candidates have demonstrated promising diagnostic potential in the literature [31,32,33,34,35]. Alpha-defensin, for instance, has exhibited excellent sensitivity and specificity—reportedly as high as 97%—in the diagnosis of PJI [32]. However, its utility in native joint infections appears to be limited, as its levels may also increase in response to non-bacterial inflammatory triggers, such as crystal-induced arthritis [34]. Calprotectin, a neutrophil-derived protein, has also gained attention due to its rapid elevation in inflammatory conditions. Studies have shown that calprotectin achieves high diagnostic accuracy in both PJI and native joint infections [31,33]. Notably, Baillet et al. reported an area under the curve (AUC) of 0.94 for calprotectin in native knee infections, outperforming alpha-defensin in their cohort [35]. Future investigations incorporating these biomarkers, in conjunction with those evaluated in our study, may further refine diagnostic algorithms and improve the differentiation between septic arthritis and other forms of inflammatory arthritis.

Our results confirmed the hypothesis that combining multiple biomarkers enhances diagnostic performance in diagnosing septic arthritis compared with a single biomarker. Previous studies on diagnostic tools for septic arthritis have relied mainly on univariate analyses to evaluate individual biomarkers [15,16,17,18,19,20]. Although identifying a single definitive biomarker remains essential, determining the optimal combination of markers may offer greater clinical utility, notably when no single biomarker demonstrates sufficient discriminatory power. A meta-analysis of 15 studies evaluating 26 biomarkers for septic arthritis proposed an algorithmic approach based on synovial WBC count, followed by individual biomarker assessments to rule in or rule out infection [9]. However, this strategy did not assess combinations of multiple biomarkers. Another study addressed this limitation by developing a multivariate analysis model incorporating clinical and laboratory findings, achieving excellent diagnostic accuracy (AUC, 0.942; *p* < 0.001) [36]. In our study, multivariate analysis outperformed univariate approaches in diagnostic performance. Combining serum CRP and synovial WBC with three novel biomarkers (PTX3, IL-6, and presepsin) yielded the highest AUC. Significantly, each biomarker contributed distinct diagnostic value when added individually. PTX3 achieved the highest sensitivity (100%) and negative predictive value (NPV, 100%), supporting its use in rule-out (screening) strategies. In contrast, presepsin showed the highest specificity (96.0%) and positive predictive value (PPV, 75.0%), indicating its strength in rule-in (confirmatory) strategies. These findings highlighted the clinical value of tailoring biomarker selection to the diagnostic objectives. In a real-world setting, septic arthritis often requires two distinct approaches: one for early screening to exclude the condition safely, and another for definitive diagnosis to guide treatment [37,38,39]. Biomarker combinations with high sensitivity and NPV are ideal for the former, while those with high specificity and PPV are preferable. Therefore, our results provide a rationale for a personalized diagnostic strategy. By selecting biomarker panels aligned with clinical decision-making needs, clinicians can improve diagnostic confidence, reduce unnecessary treatments, and enhance patient outcomes. This approach may serve as the foundation for developing future clinical decision-support tools.

This study has several limitations. First, the number of patients diagnosed with septic arthritis was relatively small, which may have limited the statistical power, particularly for evaluating individual novel biomarkers such as IL-6 and presepsin. However, despite the limited sample size, the diagnostic combinations of routinely used and novel biomarkers demonstrated consistent and clinically meaningful trends. These findings suggest that even with a modest sample size, the strategic combination of biomarkers may enhance diagnostic accuracy and support clinical decision-making in distinguishing septic from non-septic arthritis. Second, more than 37 novel biomarkers have been proposed for diagnosing septic arthritis [16]. Among the biomarkers, only PTX3, IL-6, and presepsin were included in our study owing to their availability at our hospital at the time of the survey. If we had included more biomarkers, such as sTNF-R2, which showed the best results in a previous study, we might have identified more optimal combinations and cut-off values for diagnosing septic arthritis [16]. Third, joint infections can be categorized into native septic arthritis and PJI according to the operative history of total joint arthroplasty. Our study excluded cases suspected of PJI because the diagnostic criteria for synovial WBC differed between native septic arthritis and PJI [40]. Consequently, our findings may not directly apply to the detection of PJI, and further research is needed to establish definitive diagnostic criteria for PJI. Finally, malnutrition has been known to be associated with adverse outcomes after orthopedic surgery [41]. However, our study did not include data related to malnutrition. If we had incorporated malnutrition-related factors such as albumin and lymphocyte count, the results might have been improved. Despite these limitations, our study provides meaningful insights into the diagnostic utility of biomarkers for septic arthritis, particularly through multivariate analysis. To enhance its clinical applicability, the proposed model may be incorporated into the early diagnostic workflow following arthrocentesis—especially in scenarios where culture results are pending or inconclusive. The combination of serum CRP, synovial WBC count, and selected biomarkers could facilitate more timely and accurate clinical decision-making regarding the likelihood of septic arthritis. This approach may help reduce unnecessary antibiotic administration and mitigate treatment delays, thereby improving patient outcomes.

## 5. Conclusions

The findings of our study demonstrated that, although routinely used biomarkers such as serum CRP and synovial WBC remain valuable in diagnosing septic arthritis, incorporating novel biomarkers, such as PTX3, IL-6, and presepsin, could enhance diagnostic accuracy. Notably, different biomarker combinations offered distinct diagnostic advantages: PTX3 provided the highest sensitivity and NPV, making it helpful in ruling out septic arthritis, whereas presepsin offered the highest specificity and PPV, supporting its role in confirmatory diagnosis. These findings suggest that biomarker selection should be tailored to the clinical context—whether to exclude or confirm the diagnosis—thereby supporting a more individualized and efficient diagnostic approach for septic arthritis in acute settings.

## Figures and Tables

**Table 1:** Baseline characteristics of the included patients with and without septic arthritis.

	Non-Septic Arthritis (N = 25)	Septic Arthritis (N = 6)	*p*-Value
Age, years	61.5 ± 14.6	45.8 ± 24.8	0.174 *
Sex (female/male), N, %	13/25 (52.0)	2/4 (50.0)	0.654 †
Height, cm	158.8 ± 10.9	168.4 ± 7.4	0.036 *
Weight, kg	62.2 ± 11.3	70.6 ± 7.8	0.114 *
BMI, kg/m^2^	24.6 ± 3.6	24.9 ± 1.8	0.777 *
Affected joint			0.420 ‡
Knee, %	22 (88.0)	6 (100)	
Ankle, %	1 (4.0)	0 (0)	
Shoulder, %	1 (4.0)	0 (0)	
Wrist, %	1 (4.0)	0 (0)	
Laterality (right/left), N, %	11/14 (78.6)	5/6 (83.3%)	0.172 †
Hypertension	12 (48.0)	3 (50.0)	1.000 †
Diabetes mellitus	7 (28.0)	2 (33.3)	1.000 †
Chronic kidney disease	1 (4.0)	0 (0)	1.000 †
Cerebrovascular disease	1 (4.0)	0 (0)	1.000 †
Rheumatologic disease	2 (8.0)	0 (0)	1.000 †
Endocrinologic disease	3 (12.0)	0 (0)	1.000 †
Hepatologic disease	0 (0)	1 (16.7)	0.194 †

Values are presented as mean ± standard deviation and number (percentage). Abbreviation: N, number; BMI, body mass index. Statistical analysis: * Mann–Whitney test, † Fisher’s exact test, ‡ Linear by linear association analysis.

**Table 2:** Comparison of laboratory findings between patients with and without septic arthritis.

	Non-Septic Arthritis (N = 25)	Septic Arthritis (N = 6)	*p*-Value *
Blood			
Hb, g/dL	12.1 ± 2.3	11.8 ± 1.6	0.955
WBC, cells/μL	9478.4 ± 3412.0	12,260.0 ± 4910.5	0.279
CRP, mg/dL	7.5 ± 7.5	18.1 ± 13.8	0.022
ESR, mm/hr	45.0 ± 17.1	41.3 ± 9.7	0.395
Albumin, g/dL	3.7 ± 0.5	4.9 ± 2.8	0.066
Uric acid, mg/dL	5.6 ± 3.0	4.9 ± 2.8	0.770
Joint fluid			
SG	1.027 ± 0.005	1.030 ± 0.005	0.148
pH	7.9 ± 0.4	7.9 ± 0.2	0.721
WBC, cells/μL	25,042.8 ± 26,784.2	72,028.6 ± 59,222.3	0.023
Neutrophil, cells/μL	22,488.2 ± 24,587.9	77,624.0 ± 64,225.6	0.031
Lymphocyte, cells/μL	3.3 ± 3.4	3.8 ± 3.6	0.314
Eosinophils, cells/μL	0.0 ± 0.2	0.0 ± 0.0	0.789
Neutrophil, %	69.9 ± 34.0	89.0 ± 7.3	0.327
Lymphocyte, %	12.5 ± 20.5	8.0 ± 12.4	0.865
Eosinophil, %	0.3 ± 1.1	0.0 ± 0.0	0.789
Pentraxin 3, ng/mL	53.6 ± 99.3	366.7 ± 618.0	0.017
IL-6, ng/mL	110.9 ± 146.0	190.7 ± 171.9	0.227
Presepsin, pg/mL	159.2 ± 516.6	1098.2 ± 1398.8	0.053

Values are presented as mean ± standard deviation. Abbreviation: N, number; Hb, hemoglobin; WBC, white blood cell; CRP, C-reactive protein; ESR, erythrocyte sedimentation rate; SG, specific gravity; IL-6, interleukin-6. Statistical analysis: * Mann-Whitney test.

**Table 3:** Diagnostic performance of single biomarkers for predicting septic arthritis.

	AUC (95% CI)	*p*-Value	Yonden Index	Cut Off	Accuracy	Sensitivity	**Specificity**	**PPV**	**NPV**
Serum CRP	0.817 (0.631–1.000)	0.021	0.550	5.2 mg/dL	71.0	100	64.0	40.0	100
Synovial WBC	0.837 (0.678–0.995)	0.012	0.593	34,200.0 cells/μL	77.4	83.3	76.0	45.5	95.0
PMN, %	0.648 (0.388–0.908)	0.303	0.320	94.5%	80.6	33.3	92.0	50.0	85.2
Pentraxin 3	0.813 (0.658–0.969)	0.019	0.680	36.8 ng/mL	74.2	100	68.0	42.9	100
IL-6	0.667 (0.391–0.942)	0.211	0.537	105.8 ng/mL	74.2	83.3	72.0	41.7	94.7
Presepsin	0.760 (0.526–0.994)	0.051	0.389	35.5 pg/mL	77.4	66.7	80.0	44.4	90.9

Abbreviation: AUC, area under curve; CI, confidence interval; PPV; positive predictive value; NPV, negative predictive value; CRP, C-reactive protein; WBC, white blood cell; PMN, polymorphonuclear leukocyte; IL-6, inleukin-6.

**Table 4:** Diagnostic performance of combined biomarkers for predicting septic arthritis.

	AUC (95% CI)	*p*-Value	Yonden Index	Cut Off	Accuracy	**Sensitivity**	**Specificity**	**PPV**	**NPV**
sWBC, PMN ^1^	0.540 (0.249–0.831)	0.764	0.253	−3.026	80.6	33.3	92.0	50.0	85.2
CRP, sWBC ^2^	0.853 (0.702–1.000)	0.008	0.640	−4.208	74.2	100	68.0	42.9	100
CRP, sWBC, PMN ^3^	0.713 (0.487–0.940)	0.110	0.440	−0.690	71.0	83.3	68.0	38.5	94.4
CRP, sWBC, Pentraxin 3 ^4^	0.873 (0.737–1.000)	0.005	0.720	−3.186	74.2	100	68.0	42.9	100
CRP, sWBC, IL-6 ^5^	0.880 (0.749–1.000)	0.004	0.600	−2.860	71.0	83.3	68.0	38.5	94.4
CRP, sWBC, Presepsin ^6^	0.873 (0.727–1.000)	0.005	0.600	−4.196	87.1	50.0	96.0	75.0	88.9
CRP, sWBC, Pentraxin 3, IL-6, Presepsin ^7^	0.887 (0.755–1.000)	0.004	0.560	−4.437	71.0	100	64.0	40.0	100
CRP, sWBC, PMN, Pentraxin 3, IL-6, Presepsin ^8^	0.819 (0.636–1.000)	0.017	0.667	2.031	83.3	83.3	83.3	55.6	95.2

Abbreviation: AUC, area under curve; CI, confidence interval; PPV; positive predictive value; NPV, negative predictive value; sWBC; synovial white blood cell; PMN; percent of polymorphonuclear leukocyte; CRP, C-reactive protein; IL-6, interleukin-6. Regression equations of the combination of biomarkers. ^1^ Equation (sWBC, PMN) = −3.687 + 0.000 × Synovial WBC + 0.007 × Neutrophil. ^2^ Equation (CRP, sWBC) = −3.795 + 0.095 × Serum CRP + 0.000 × Synovial WBC. ^3^ Equation (CRP, sWBC, PMN) = −3.190 + 0.081 × Serum CRP + 0.000 × Synovial WBC + (−0.006) × Neutrophil. ^4^ Equation (CRP, sWBC, PTX3) = −3.899 + 0.093 × Serum CRP + 0.000 × Synovial WBC + 0.002 × Pentraxin 3. ^5^ Equation (CRP, sWBC, IL-6) = −6.699 + 0.144 × Serum CRP + 0.000 × Synovial WBC + 0.008 × IL-6. ^6^ Equation (CRP, sWBC, Presepsin) = −5.023 + 0.133 × Serum CRP + 0.000 × Synovial WBC + 0.002 × PP. ^7^ Equation (CRP, sWBC, PTX3, IL-6, Presepsin) = −6.621 + 0.154 × Serum CRP + 0.000 × Synovial WBC + (−0.001) × Pentraxin 3 + 0.006 × IL-6 + 0.001 × Presepsin. ^8^ Equation (CRP, sWBC, PMN, PTX3, IL-6, Presepsin) = 2.981 + 0.232 × Serum CRP + 0.000 × Synovial WBC + (−0.094) × Neutrophil + 0.000 × Pentraxin 3 + 0.014 × IL-6 + 0.001 × Presepsin.

## Data Availability

The data presented in this study are available on request from the corresponding author due to institutional data protection policies.

## Abbreviations

AUC	area under the curve
BMI	body mass index
CRP	C-reactive protein
IL-6	interleukin 6
PJI	periprosthetic joint injection
PMNs	polymorphonuclear leukocytes
PTX3	pentraxin 3
sTNF-R2	soluble tumor necrosis factor receptor 2
WBCs	white blood cells
